# Insulin-like growth factor family and prostate cancer: new insights and emerging opportunities

**DOI:** 10.3389/fendo.2024.1396192

**Published:** 2024-05-30

**Authors:** Noha M. Elemam, Hassan Youssef Hotait, Mohamed A. Saleh, Waseem El-Huneidi, Iman M. Talaat

**Affiliations:** ^1^ Clinical Sciences Department, College of Medicine, University of Sharjah, Sharjah, United Arab Emirates; ^2^ Research Institute for Medical and Health Sciences, University of Sharjah, Sharjah, United Arab Emirates; ^3^ Pathology & Genetics Department, Dubai Hospital, Dubai, United Arab Emirates; ^4^ Department of Pharmacology and Toxicology, Faculty of Pharmacy, Mansoura University, Mansoura, Egypt; ^5^ Basic Medical Sciences Department, College of Medicine, University of Sharjah, Sharjah, United Arab Emirates; ^6^ Pathology Department, Faculty of Medicine, Alexandria University, Alexandria, Egypt

**Keywords:** prostate cancer, IGF-I, IGF-II, IGF-1 receptor, IGF-2 receptor

## Abstract

Prostate cancer is the second most commonly diagnosed cancer in men. The mammalian insulin-like growth factor (IGF) family is made up of three ligands (IGF-I, IGF-II, and insulin), three receptors (IGF-I receptor (IGF-1R), insulin receptor (IR), and IGF-II receptor (IGF-2R)), and six IGF-binding proteins (IGFBPs). IGF-I and IGF-II were identified as potent mitogens and were previously associated with an increased risk of cancer development including prostate cancer. Several reports showed controversy about the expression of the IGF family and their connection to prostate cancer risk due to the high degree of heterogeneity among prostate tumors, sampling bias, and evaluation techniques. Despite that, it is clear that several IGF family members play a role in prostate cancer development, metastasis, and androgen-independent progression. In this review, we aim to expand our understanding of prostate tumorigenesis and regulation through the IGF system. Further understanding of the role of IGF signaling in PCa shows promise and needs to be considered in the context of a comprehensive treatment strategy.

## 1 Introduction

Prostate cancer (PCa) is the fifth leading cause of cancer-related mortality in men worldwide, as well as being the second most commonly diagnosed solid-organ cancer, after lung cancer, in men (1, 2). PCa happens at a rate of 11.3 per 100,000 in developing countries and 37.5 per 100,000 in industrialized countries (3). Similarly, mortality rates in developed and developing nations are 8.1 and 5.9 per 100,000, respectively. According to current estimates, approximately 10 million males are presently diagnosed with PCa, with approximately 400,000 deaths per year, and this figure is expected to rise to over 800,000 by 2040 (3, 4). Although PCa is generally diagnosed at an early stage, the risk-benefit ratio of the treatment remains uncertain. It still represents a global challenge because of the significant morbidity from the current form of therapy and the long disease history and uncertainty in individual patients’ clinical progress (5–7). Approximately 5% of men diagnosed with PCa are diagnosed with distant metastases (often in multiple sites), and 15% are diagnosed with locoregional metastases (8). Such cases have a poor overall survival rate of only 30% for five years (8).

## 2 The insulin-like growth factor family

The mammalian insulin-like growth factor (IGF) family is made up of three ligands (IGF-I, IGF-II, and insulin) and three receptors (IGF-I receptor (IGF-1R), insulin receptor (IR), and IGF-II receptor (IGF-2R)) (9). IGF-1R and IR are receptor tyrosine kinases (RTKs) that are structurally similar hetero-tetramers. IR has two alternatively spliced isoforms, IRA and IRB, whose functions are currently unknown. IGF-II, which binds to IRA and IGF-1R and is a more potent mitogen than IGF-I, has recently been demonstrated to control IR isoforms rather than insulin-binding affinities (10). IRB governs metabolic processes in adults, whereas IRA controls prenatal growth and development and mediates the mitogenic effects of insulin. A family of six IGF-binding proteins (IGFBPs) tightly controls the amounts of IGF-I and IGF-II as well as their bioavailability in the circulation and cells (11). IGFBPs are distinct from ligands and receptors and have a greater affinity (pM) for IGFs than their corresponding receptors (nM) (10). IGF-I and IGF-II are produced by a variety of cells, including the liver and muscles, among others. They are secreted constitutively as opposed to being retained in the cells of origin, where they serve as paracrine/autocrine factors (12).

### 2.1 IGFs

In mammalian cells, the production of IGF-I is mainly induced by growth hormones and transcriptional factors. Then it was shown that IGF-II (67 amino acids) has comparable growth-promoting properties but growth hormone does not regulate its expression (13–15). Additionally, IGF-I and IGF-II are quite similar to insulin in terms of amino acid sequence (16). The three disulfide connections that are shared by these three peptides, allow them to preserve the proper peptide shape. IGFs differ structurally from insulin as they are composed of single chains with a connecting domain (C domain) between the N-terminal B chain and the C-terminal A chain. They result from the elimination of the N- and C-terminal signal peptides from pre-pro-IGF peptides during post-translational processing. The chaperone GRP94 aids in the folding and maturation of the IGF peptides (17, 18).

Studies using knock-out and transgenic mice have revealed additional details about the biological actions of mammalian IGFs. *Igf1-null* mice had a high postnatal mortality rate and were 30% smaller and lighter than wild-type mice (19). The postnatal growth retardation was also present in those who survived, and it was particularly noticeable during both growth stages (pubertal and post-pubertal). Their bones grew more slowly, and their organs were proportionately smaller. *Igf-1r* knockout also caused a serious growth deficit and was embryonically fatal in mice (19).

Despite the findings that unraveled the potential role of IGF-I and IGF-II in development, it is still reported that the expression of both IGF-I and IGF-II is tissue-dependent and time-dependent (20). On the other hand, the role of IGF-II in physiology and disease has been the subject of far fewer investigations than that of IGF-I. However, like IGF-I, most tissues in adults produce IGF-II, with the liver producing the majority of the circulating levels. Notably, IGF-II levels in adults are roughly three times higher than IGF-I. Despite this, IGF-II is thought to play crucial roles in fetal growth and development, and it is abundant in the fetal skeletal muscle (12, 21). The stimulation of the IRA to promote stem cell self-renewal is another unique action of IGF-II. Moreover, the expansion of neural progenitor cells and neural stem cell maintenance is supported by IGF-II/IRA signaling (22).

### 2.2 IGFBPs

It was evident that the preponderance of circulatory IGFs was much larger than the concentration of peptides in the bloodstream because IGFs were not bound to binding proteins (23). In humans and other mammals, six highly similar high-affinity IGF-binding proteins (IGFBP-1 to IGFBP-6) were identified (24). IGFBPs are pluripotent and used in a variety of metabolic processes. All IGFBPs have conserved three subdomains, including high-affinity IGF-binding terminal domains. Contrarily, one other unstructured domain (known as the central linker domain), is thought to be responsible for the various functions that are unique for each IGFBP (25). Furthermore, it has been reported that the specific function of IGFBP is affected by many post-translational modifications (such as proteolytic cleavage, glycosylation, and phosphorylation). Although this division may not be rigorous, it is possible to roughly divide the functions of IGFBPs into those that rely on their ability to bind and control the activity of IGFs and those that appear to be independent of direct IGF binding (26, 27).

Due to the higher affinity of IGFBPs for IGF-I and IGF-II, the availability of free IGFs decreases and this inhibits them from binding and activation of their receptors. IGFBPs have two key functions that are both critical to the metabolism of IGFs (28). IGFs are produced and swiftly secreted via the constitutive secretory route, as tissues do not contain any intracellular storage of IGFs despite their widespread distribution throughout the body. As soon as they are secreted, IGFs bind to high-affinity IGFBPs, creating binary complexes of about 30–40 kDa. IGFs bound to two IGFBPs, IGFBP-3 and IGFBP-5, can join with the acid-labile subunit (ALS), a third glycoprotein, to form a ternary complex that is about 150 kDa in size (29). Without the ability to store them in tissues, the body can build up enormous IGF reservoirs due to IGFBPs. In humans, the total amount of IGFs in circulation is about 100 nM, with 80–90% of that amount being found in the ternary complex with IGFBP-3 (30). The second important function of IGFBPs is the creation of new pathways for giving IGFs specificity. IGFs are made in most tissues and can regulate the bulk of cell processes. IGFBPs are synthesized in numerous tissues, at different times, in various amounts, and in several combinations to add some specificity to the IGF activity (12).

Furthermore, at the cellular level, IGFBPs can boost IGF activity in several different ways. This can happen by altering the kinetics of IGF ligand/receptor interactions and preventing receptor downregulation, or by changing the interactions between IGFBPs and ECM or cell surfaces thus localizing and increasing the IGF concentrations near to cell receptors (31).

### 2.3 IGF receptors

Both IGF-1R and IR are synthesized as polypeptide precursors, which are then modified post-translationally, where α and β subunits are formed upon the cleavage of the precursor molecule. The heterotetramer receptor is made up of two α and two β subunits that are joined together by disulfide bridges. 627 amino acids make up the IGF-1R -subunit, 196 of which are found in the extracellular domain. The intracellular and extracellular domains are joined by a brief transmembrane domain (TM). The juxtamembrane domain (JM), enzymatic tyrosine kinase (TK) domain, and C-terminal domain are the three subdomains of the β-subunit’s intracellular domain. Positions 976 to 981 are occupied by the TK ATP-binding motif (GXGXXG), while position 1003 contains a catalytic lysine that is essential for Mg-ATP binding. The activation loop of the TK domain contains a trio of tyrosines at positions 1131, 1135, and 1136 that are crucial for receptor autophosphorylation. The JM region contains an NPEY motif that, after being phosphorylated, serves as a docking site for Shc and the insulin receptor substrates (IRS), whose recruitment signifies the start of the downstream signaling process. The internalization of receptors, which controls signaling, depends on the NPEY motif (32, 33).

Generally, the IGF-1R, which is expressed on most cells, plays specialized roles in well-differentiated cells such as neurons as well as being involved in cellular proliferation and anti-apoptosis during growth and development. The IGF-pluripotent IR’s roles, however, are still being defined; for instance, a novel function for the IGF-IR in viral entry into cells has just been identified (34).

Several tissue-growth-related transcription factors, including androgen and estrogen receptors, high-mobility group A1 (HMBA1), Krüppel-like factor 6 (KLF6), eukaryotic translation initiation factor 2 (E2F1), and c-Jun, upregulate IGF-IR expression (12, 35). In contrast, several tumor-suppressor genes, including p53, WT1, and BRCA1 downregulate it (36–38).

## 3 Post-receptor signaling

The binding of IGF-I, IGF-II, or insulin ligand to the IGF-1R initiates a series of events. The binding of IGFs to their receptors activates both MAPK and PI3K-AKT pathways, which results in downstream cellular effects (such as cellular proliferation, anti-apoptosis, and differentiation actions) through other downstream molecules (39). Furthermore, IGF-1R signaling could be mediated through several proteins such as JNK, Jak1, Jak2, focal adhesion kinase, and TIMP2 (40). Moreover, crosstalk between IGF-1R and G-protein coupled receptors (GPCRs) has been reported which may indicate a potential role of IGF-1R in cancer.

IRS 1-4 and other signaling proteins, such as Shc, bind to the IGF-1R at the extracellular subunit as a result of the binding of IGF-I and IGF-II (41). This autophosphorylation of the subunit residues prepares it to serve as a docking site for these proteins. PI3(p110) kinase’s subunit catalyzes the recruitment of protein kinase B (Akt) to the cell membrane (42), causing its phosphorylation and activation, then PI3 regulatory kinase’s component, p85, is recruited by IRS-1. Bcl2 antagonist of cell death (Bad), glycogen synthase kinase 3 (GSK3), forkhead transcription factors (FOXO1), and Akt substrate of 160 kDa are only a few of the many substrates for activated Akt (AS160). These elements have a major role in controlling cell metabolism and apoptosis (42, 43). By phosphorylating the protein Tuberous Sclerosis Protein (TSC2), Akt controls protein synthesis by loosening its inhibition on Rheb and activating the mammalian target of rapamycin (mTORC1). A set of mitogen-activated protein kinase (MAPKKK, MAPKK, and MAPK) pathways can be initiated by the phosphorylation of IRS-1 and Shc, which can also result in the recruitment of Grb2, SOS, and Ras. These pathways can then be activated, which promotes cell growth, migration, and survival (44, 45).

Recent research has shown that the IGF-1R (and IR) can migrate to the nucleus in both healthy and cancerous cells through mechanisms that are still being fully elucidated, although sumoylation is thought to be one method. The IGF-1R can bind to DNA and control the transcription of its own receptor gene as well as genes involved in apoptosis and the cell cycle. A more thorough analysis of the whole range of IGF-1R effects within the cell was completed (46).

The internalization of the IGF-1R is a complicated process that includes subcellular transport, intracellular signaling, and recycling of the receptor to the surface in addition to partial destruction of the receptor. The ligand binding process starts with the IGF-increased IR’s internalization (endocytosis). Through substrates that specifically bind to tyrosine residues 1250 and 1251 in the C-terminus, internalization, and degradation play a part. Internalization separates the ligand from the receptor via the acidic endosomal route, where caveolin- or clathrin-dependent mechanisms may be used for internalization (47). The lysosomal or proteasomal routes are both options for the receptor’s degradation. The process of receptor ubiquitination, which is brought on by ligand interaction, results in the receptor’s destruction by the proteasome. The receptor concentration on the surface may therefore be downregulated as a result of ligand binding and internalization, even though the levels may be adjusted by increased IGF-1R gene expression. A collection of proteins called adhesion-associated proteins may regulate subcellular transport. These include the discoidin domain receptor 1 (DDR1), non-receptor tyrosine adhesion kinase FES-related (FER), and non-integrin collagen RTK. The IGF-1R can translocate to the nucleus or the Golgi apparatus’ internal membrane compartments for destruction (28, 47). Intracellular signaling, which appears to be important in the migratory behavior of cancer cells, can be started by the IGF-1R in the Golgi. IGF-I induces the translocation of IGF-1R to the nucleus, where it may bind with DNA to increase transcription, with consequences that seem to support an aggressive cancer phenotype (28, 47).

## 4 The role of the IGF family in carcinogenesis

**Figure 1:**
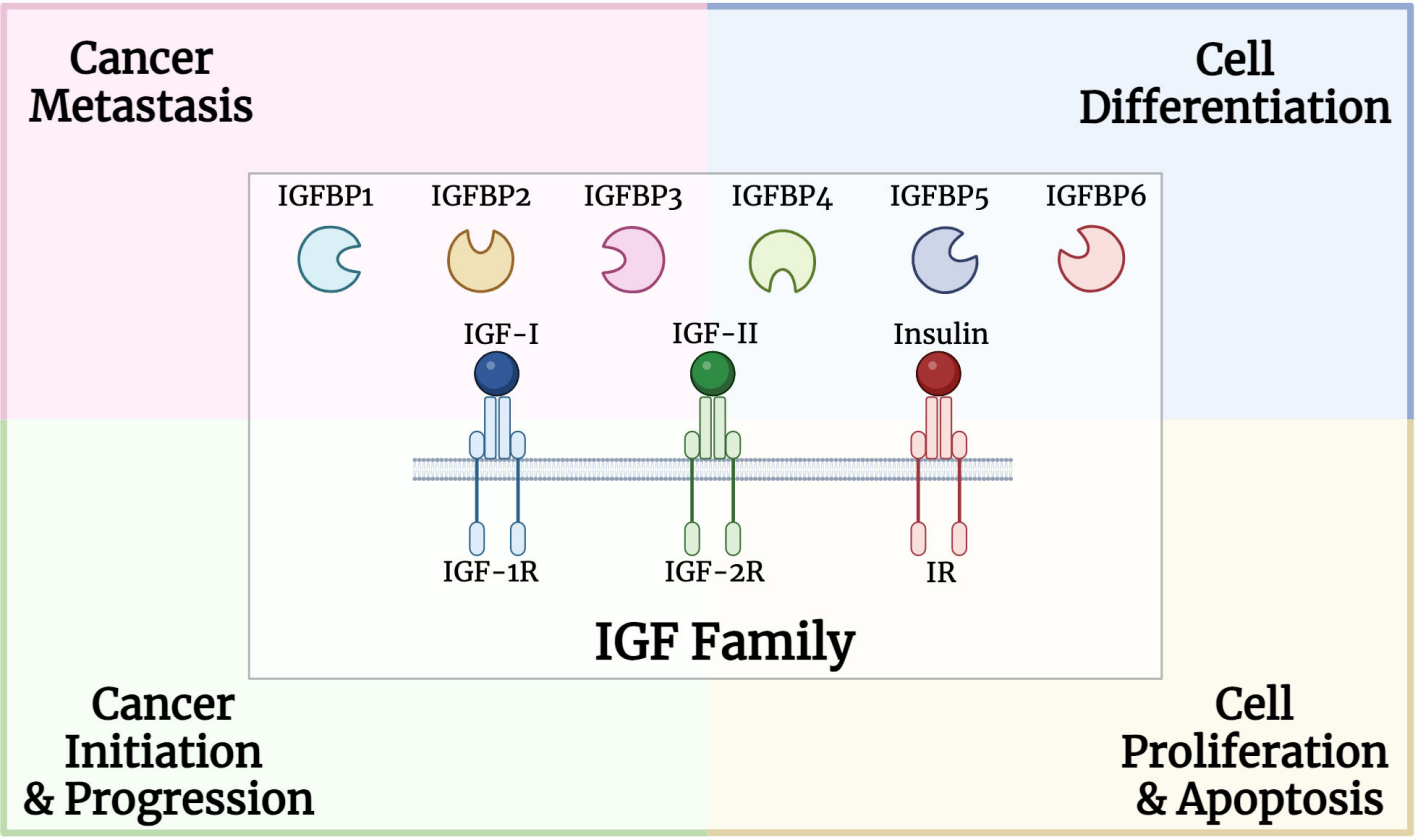
Structure and role of insulin growth factor (IGF) family, including binding proteins, ligands, and receptors, in multiple processes involved in carcinogenesis.

The IGF family plays a critical role in various cellular processes such as proliferation, differentiation, and apoptosis (**Figure 1**). In particular, IGFBPs protect IGFs from degradation and regulate their interactions with the receptors. High circulating levels of the potent mitogen, IGF-I, were previously associated with an increased risk for breast, prostate, lung and colorectal cancers (48–51). Both mitogens, IGF-I and IGF-II, were identified to be overexpressed in various cancer types such as sarcoma, leukemia, breast, lung, colon, stomach, esophagus, liver, pancreas, kidney, thyroid, brain, ovary, cervical, endometrial and prostate cancers (52–57). High IGF-IR expression was positively associated with worse disease outcomes for several cancer types, including prostate and gastric cancer, as well as renal cell carcinoma (58–60).

IGF-I, the crucial peptide hormone involved in controlling human growth and development, functions by promoting cell growth and preventing apoptosis (54, 61, 62). Such actions have a significant impact on the tumor development (63). Other members of the IGF family directly affect cancer-related cellular activities and interact with a wide range of molecules that are crucial for cancer initiation and progression. IGF-I has been associated with the activation of the MAPK and PI3K signaling pathways (64). Additionally, diet and exercise have an impact on the expression and production of IGF-I (65). IGF-I overexpression was previously identified to induce tumor development (66, 67), while high expression of IGF-1R and IGF-II triggered cancer metastasis (68). Additionally, IGF-1R was reported to be essential for cell transformation triggered by oncogenes and tumor-virus proteins (53). Several approaches were implemented to inhibit the mitogenic effect of IGF signaling including eliminating IGF-1R from the cell membrane, blocking the interaction of IGFs with IGF-1R, or interrupting the signal transduction pathway (53, 69–71). On the other hand, IGF-2R is known to antagonize the effect of IGF-II, where tumors that had low expression of IGF-2R or possessed IGF-2R mutations and could not hence degrade IGF-II, had much higher growth rates (72, 73). Thus, restoring IGF-2R expression induced apoptosis and reduced the growth of cancer cells (74).

IGFBPs regulate the interaction between IGF-I and IGF-1R and affect the mitogenic activity of IGF-I (75–84). One of the most studied IGFBPs is IGFBP-3 which prolongs the half-life of the IGFs and regulates their availability to the cell surface receptors (85, 86). Besides, IGFBP-3 has anti-proliferative actions affecting cancer growth (87, 88). It was previously reported that serum levels of IGFBP-3 were inversely associated with cancer risk in those patients. Also, IGFBP-3 was found to inhibit breast and prostate cancer growth and induce apoptosis (89–91). Moreover, vitamin D and its synthetic analogs could increase the expression of IGFBPs and reduce IGF-1R and IGF-II in breast and prostate cancer (92–95). Another factor affecting the expression of IGF family members is the expression of tumor suppressor genes. For example, wild-type p53 protein induces IGFBP-3 expression, represses the transcription of IGF-II, and suppresses IGF-1R expression (96–101).

## 5 The impact and expression of the IGF family in neoplastic prostate cells and tissues

During the transition from benign to malignant state in prostate cancer, several IGF family members change. For instance, IGF-I, IGFBP-2 and IGFBP-5 levels rise, while IGF-IR and IGFBP-3 levels lessen (82, 102–104). The controversial reports about the expression of the IGF family and its connection to prostate cancer risk and development may be the result of the high degree of heterogeneity among prostate tumors, sampling bias, evaluation techniques, or the experimental design (105).

Some proteases produced in the prostate cancer microenvironment were previously identified to enhance IGF-I/IGF-1R signaling. These include prostate-specific antigen (PSA), human kallikrein 2, trypsin, and cathepsin D, which were identified to degrade IGFBPs, thus releasing free mitogenic IGF-I (106, 107). High IGF-I expression and low IGFBP-3 in prostate cancer were identified as contributors to cancer initiation and progression by affecting cellular transformation, apoptosis, and metastasis (108). IGF-I and IGFBP-3 expression in prostate cancer patients was strongly associated with more advanced or aggressive disease, indicating a critical role in cancer progression (49). Further studies highlighted the potential of IGF-I as a strong predictor of advanced rather than early-stage prostate cancer (109). On the contrary, a study by Ma et al. reported no significant associations between free IGF-I and other IGF-I biomarkers with lethal and non-lethal prostate cancer (110). However, blood levels of IGFBP-3 showed contradictory results in prostate cancer (111). Serum levels of IGF-I, IGF-II, IGFBP-2, IGFBP-4, and IGFBP-5 were found to be higher in prostate cancer patients (82, 103, 112–116). Moreover, IGFBP-2 expression was found to be higher in the prostate cancer tissue compared to the normal tissue counterpart (102). Neither IGF-II nor IGFBP-2 concentrations were associated with prostate cancer risk (117). On the other hand, low levels of IGFBP-3 were reported in the serum and prostate tumor tissue of cancer patients (102). Moreover, IGF-I was found to be significantly elevated while IGFBP-3 was reported to be reduced in prostate cancer tissues (118). Also, such an expression was found to be linked to tumor size and hyperplasia (118, 119). IGF-I and IGFBP-3 plasma levels were suggested to be indicators of prostate cancer risk (112). Moreover, the lower IGFBP-3 levels could lead to enhanced IGF-I bioavailability (85). In addition, the plasma levels of IGFBP-3 were considerably lower in African-American men compared to white men (120, 121). This could support the findings that African-American men have a higher incidence of prostate cancer than white men (122). Additionally, high IGF-I levels in the blood/serum of healthy men were associated with a high risk of developing prostate cancer (123, 124). This suggests that prolonged exposure to high concentrations of IGF-I could trigger carcinogenesis of prostate epithelial cells (125). Therefore, targeting IGF-I could be a potential therapeutic approach in prostate cancer.

When compared to the benign prostate epithelium, primary prostate cancer had higher levels of IGF-1R expression, which is further escalated in metastasis (126). This was further supported by studies using human prostate xenografts where higher IGF-1R expression was found in metastatic and androgen-independent tumors (127, 128). Also, transgenic mice with a prostate-specific deletion of IGF-1R and the tumor suppressor gene p53 had more aggressive prostate cancer than their wild-type counterparts (129).

*In vitro*, studies indicated that IGF-I stimulated the proliferation of various prostate cancer cell lines (22Rv1 and DU145) by the activation of the AKT/ERK/MAPK pathway (130). Also, IGF-I regulated the invasion potential of DU145 prostate cancer cells by controlling the activity of MMP-2 and MMP-9 as well as secreted TIMP-2 levels, that are transduced via the PI3K and MAPK pathways (131). Further, IGF-I was described to regulate the expression of miR-143, leading to an increase in IGFR expression in PC-3 and DU145 prostate cancer cell lines. Such an effect was found to lead to resistance to docetaxel treatment (132). Additionally, it has been demonstrated that IGF-I activated androgen receptor signaling in prostate cancer cells via the IGF-1R-forkhead box protein O1 (FOXO1) signaling axis, which is also implicated in castration-resistant prostate cancer (133–135). *In vivo*, PC-3 tumors proliferate at a considerably slower rate in IGF-I-deficient hosts than in IGF-I-expressing hosts (136). Mice injected with the androgen-sensitive and PSA-producing LNCaP cell line were found to develop tumors and their serum PSA levels were correlated with tumor volume (137). Also, mice fed a low-fat diet with xenografts of LAPC-4 showed a decrease in tumor size along with a reduction in the IGF-I expression (138). Other *in vivo* studies reported that the blocking of IGF-1R in combination with castration inhibited prostate cancer growth (139, 140). However, the most thoroughly studied IGF-1R inhibitor, limsitinib, was explored in a phase II study, where it did not significantly alleviate prostate-specific antigen levels after 12 weeks of treatment or increase the overall survival in men with metastatic castrate-resistant prostate cancer (141).

IGFBP-3 is the most prevalent form of the IGFBPs, which has been linked with prostatic growth. The majority of serum and prostatic IGF-I binds to IGFBP-3, thus regulating its concentrations (142). IGFBP-3 was identified to have anti-tumor effects by regulating multiple processes such as adhesion, motility, proliferation, and invasion of prostate cancer cells (143). Additionally, *in vitro* studies using PC3 or DU145 cell lines showed that IGF-I regulates cell adhesion and motility that is needed for the formation of a pre-metastatic niche (144). This was reported to be mediated through integrin expression especially α3, α5, and β1 expression pattern and distribution (145).

The second most prevalent IGFBP is IGFBP-2, which has a growth inhibitory effect on healthy prostate epithelial cells but a strong stimulatory effect on prostate cancer cells via the activation of MAPK and PI3K pathways (146). Also, high IGFBP-2 levels in prostate cancer patients not receiving neoadjuvant hormonal therapy had worse survival compared to patients with low IGFBP-2 levels, thus indicating an androgen effect on the IGFBP-2 action (147).

Proteases targeting IGFBPs are known to degrade IGFBPs into small fragments, thus reducing the affinity of IGFBPs to IGFs. Prostate-specific antigen (PSA) is known to be an IGFBP protease while γ-nerve growth factor (NGF) is also known to degrade IGFBPs 3, 4, 5, and 6, thereby boosting IGF action (11). Furthermore, other proteases such as human kallikrein 2 (hK2), trypsin, MMPs, and cathepsin D, present in the prostate tumor microenvironment were identified to affect IGFBP-3 and release free IGF-I (107). Protease-resistant IGFBPs could be a potential therapeutic agent in cancer therapy by preventing the mitogenic activity of IGF. Also, it was interesting to find that inhibiting the signaling pathway of epidermal growth factor (EGF), had a suppression effect on IGF-I in prostate cancer cells (148). The protease PAPP-A is responsible for the cleavage of IGFBPs 2, 4, and 5 (149), which was previously linked to cancer development in various types including prostate cancer (150–155). A possible explanation for the role of PAPP-A is through increasing the levels of IGFs and their downstream signaling pathway (156). Studies have reported that PAPP-A levels were elevated in prostate cancer patients, especially those with metastasis (157). On the other hand, several studies reported that the protease-resistant IGFBP-4 led to an inhibition of cell growth and angiogenesis (155, 158). Other *in vitro* and *in vivo* studies indicated that the mutant IGFBP-2 was able to inhibit tumor growth possibly by inhibition of angiogenesis (159).

A subgroup of IGFBPs is called IGFBP-related proteins (IGFBPs-rP) also termed the CNN family (124). These IGFBP-rPs could be critical for the regulation of stromal and epithelial cell growth in the prostate. An interesting protein is insulin-like growth factor binding protein-related protein 1 (IGFBP-rP1)/IGFBP-7. It was found to possess tumor suppressor effects through inhibiting cell growth, and triggering apoptosis and senescence (160). In contrast, another study indicated that it could promote glioma cell growth and migration (161). IGFBP-7 resulted in the activation of the translational repressor 4E-binding protein 1 (4E-BP1) and triggered apoptosis in IGF-1R^+^ cells. It suppressed IGF-1R downstream signaling, hence hindering protein synthesis, cell growth, and survival (162). *In vitro* and *in vivo* studies revealed a downregulation of IGFBP-5 in prostate cancer cells that inhibited IGF cell growth. In benign prostatic hyperplasia (BPH), IGF-II and IGF-1R were found to be overexpressed by stromal prostate cells. Also, these cells expressed IGFBP-5 which is identified to potentiate the IGF actions (163). Reduced expression of IGFBP-rP1 is associated with carcinogenesis, especially in highly tumorigenic and metastatic prostatic cells (164). IGFBP-rP1 functions as a tumor suppressor in prostate cancer cells, as its overexpression slowed down growth and proliferation rates (165). Also, prostatic cell growth is regulated by IGFBP-rP2 and IGFBP-rP3. Several factors can affect the expression of IGFBP-rP2. For instance, TGF-β boosts the expression of IGFBP-rP2 in both normal and cancerous prostate cells while IGF-I could decrease its expression. IGFBP-rP3 may act as a growth stimulator for prostate cancer cells given that it is preferentially expressed in malignant cells (166). CyrH61/IGFBPs-rP4 is localized in the mesenchyme of the benign prostatic tissue and is downregulated in prostate cancer tissues as well as in cancer cell lines (124, 167).

Our understanding of prostate tumorigenesis and regulation has been expanded especially due to our understanding of the IGF system. A high IGF-I to IGFBP-3 ratio was linked with an increased risk of prostate cancer and could be used as a predictor for prostate cancer development (168). On the other hand, another study by Saleh SAK et al. reported that changes in the serum levels of IGF-I and IGFBP-3 were not considered pre-diagnostic risk factors for prostate cancer development (142). Besides, serum levels of total PSA and free/total PSA ratio were found to be higher in prostate cancer patients (142). Also, PSA and IGF-II showed the power of prognosis and differentiation between prostate cancer and BPH (169).

## 6 The link between IGF and prostate cancer metastasis

**Figure 2:**
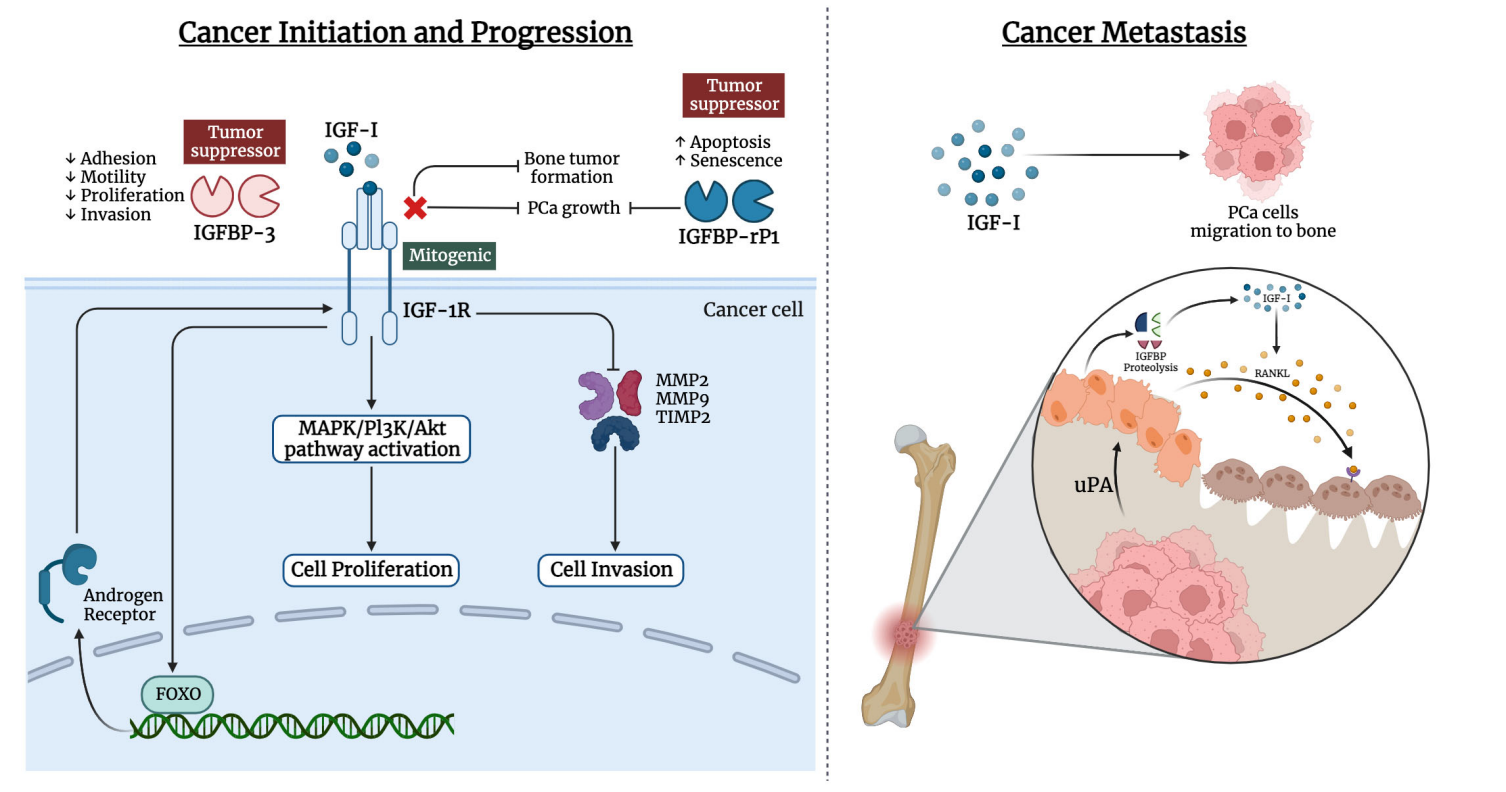
Involvement of IGF ligands, receptors, and binding proteins in prostate cancer. The left panel illustrates the interplay of IGF-I in cancer initiation and progression of prostate cancer, while the right panel highlights the IGF-I effect in metastasis of prostate cancer cells to bone tissue.

Bones are the most frequent metastatic site for prostate cancer, with 70–80% of patients experiencing skeletal metastases. Prostate cancer can metastasize to numerous organs, including the liver, lymph nodes, lung, and bone. The vertebral column, ribs, skull, and proximal ends of the long bones are among the well-vascularized parts of the skeleton where prostate cancer cells spread frequently. IGF-I and IGF-II, as well as type I and type II IGF receptors, are expressed by bone cells (170). High levels of IGF-I in the primary tumor environment seem to encourage cancer cells to spread to the bone *in vivo*. Moreover, prostate cancer cell lines that highly expressed IGF-1R were likely to develop larger bone mass (171–173). Notably, IGFs influence cell-cell adhesion and motility of cancer cells through the integrin system such as E-cadherin (174–176). The likelihood of metastasis is influenced by such interactions (177–179). Prostate cancer cells can release mediators that change the balance between osteoblast and osteoclast activities, leading to osteoblastic metastases. IGF-I plays a critical role in bone formation and bone resorption through the receptor activator of the nuclear factor-B ligand (RANKL) system in bone (180). IGF-I upregulates the expression of RANKL by bone marrow stromal cells, and osteoblasts, which can bind to RANK on the surface of osteoclast precursors. This ligand-receptor interaction activates NF-κB, which stimulates the differentiation of osteoclast precursors to osteoclasts (180–183). Also, metastatic prostate cancer cells release a urokinase-type plasminogen activator that binds to the corresponding receptor on the surface of osteoblasts. This could trigger the proteolysis of IGFBPs and increase in the bioavailability of IGFs, thus stimulating the proliferation of osteoblasts and cancer cells (**Figure 2**). A study by Goya M. et al. reported the potential of KM1468, a monoclonal antibody against IGF-I and IGF-II that inhibited the development of new bone formation and the progression of existing bone tumors (184). Also, IGF-I is secreted by the liver, which attracts circulating cancer cells drain and promotes colonization, proliferation, and the establishment of metastasis (185).

IGF-1R was found to control lymphatic metastasis of cancer by inducing the production of VEGF, thus promoting angiogenesis and lymphangiogenesis (186). This was supported by high levels of VEGF in prostate cancer patients with lymph node metastases (187). Androgen deprivation activated FOXO-1 and upregulated VEGF production by inhibiting the IGF-1R pathway (188). By activation of the IGF-1R/Akt/NF-κB pathway, bone-derived IGF-I is the link between metastasized cancer cells during bone metastasis (189).

## 7 The relation between IGF family and androgen-independent progression

Prostate cancer could progress to be castration-resistant. Several mechanisms have been put forth to explain the progression including: 1) constitutive activation of the androgen receptor due to alternative splicing of the androgen receptor; 2) epigenetic mutations of the androgen receptor; 3) transactivation of the androgen receptor by growth factors and cytokines; and 4) intracrine steroidogenic pathways within the prostate tumor (190–193).

Another critical aspect of the IGF family is that they play a role in the progression of androgen-independent prostate cancer by promoting the growth and survival of prostate tumor cells. Alternative stimulatory pathways that were activated after androgen withdrawal, were found to trigger intracellular signal transduction pathways by completely avoiding or activating the androgen receptor. For example, non-ligand activation of androgen receptors could be initiated by IGF-I, MAPK, and AKT contributing to cell proliferation in an androgen-depleted environment (194). Also, prostate cancer mouse models showed progression from androgen-dependence to androgen-independence to be associated with an increase in IGF-I and decrease in IGFBP-3, further suggesting their role in cancer aggressiveness (128). In mice, castration induced upregulation of IGFBP-5 that enhanced the development of androgen independence (195).

The effective treatment for patients with advanced prostate cancer is androgen ablation, leading to tumor regression (195). This is because proliferation of prostate cancer cells is dependent on androgens. The androgen receptor is known to bind to testosterone and dihydrotestosterone, after which a conformational change and interaction with specific DNA elements occur. The development of androgen-independent prostate cancer takes place as a consequence of the lack of apoptosis that triggers tumor cell survival (196). Furthermore, other mechanisms are associated with the development of androgen-independent prostate cancer including an increase in the sensitivity of the androgen receptor by coactivators as well as enhancement of the signaling pathways by other growth factors (197, 198). Unlike androgens, IGF-I activates tyrosine kinase-associated surface receptors, leading to the proliferation of prostate cancer cells. This is besides the effect of IGF-I on enhancing the androgen receptor transcriptional activity and its sensitivity to suboptimal stimulation by low androgen levels (199). Metastatic and androgen-independent prostate cancer specimens exhibited an increase in IGF-1R expression (126–128). On the other hand, other studies indicated that reduced IGF-1R expression is required to induce androgen independence, proliferation, and metastasis (200, 201). It was suggested that dysregulation of IGF-1R expression through KLF6 loss-of-function may be an intrinsic mechanism for prostate cancer progression to hormone independence. Also, the tumor suppressor BRCA1 was identified to interact with the androgen receptor in prostate cancer cells and regulate IGF-1R production (202). Furthermore, BRCA1 can suppress IGF-1R promoter activity in androgen receptor-negative prostate cancer cell lines but enhance IGF-1R expression at the transcriptional level in androgen receptor-positive prostate cancer cell lines (203).

Also, long-term androgen suppression may boost the resistance of prostate cancer cells to apoptosis through the inhibition of PI3K/AKT pathway (204). IGF-1R expression can be enhanced by androgens via the activation of the ERK pathway (205). Neuropeptides, including endothelin-1, vasoactive intestinal peptide, and neurotensin, were previously identified to boost IGF signaling and stimulate androgen-independent prostate cancer (111, 206–208). Moreover, castration or anti-androgen treatment induces the expression of IGFBP-2,3,4 and 5 (209, 210). This could be due to an adaptative mechanism to enhance IGF-I mediated mitogenesis, thus leading to androgen independence progression (195, 195, 211). Also, prostate cancer samples and cell lines exhibited growth inhibition when treated with diethylstilbestrol (DES) (212). The expression of the growth-promoting IGFBP-6 was reported to be induced by DES treatment, thus, highlighting its contribution to the effect of DES in androgen-independent prostate cancer (213). Pre-clinical evidence supported the utilization of insulin/IGF-targeting agents in combination with androgen deprivation therapy for prostate cancer. This is attributed to the effect of insulin on the production of androgens by prostate cancer cells, which accelerated the emergence of castration-resistant prostate cancer (214).

## 8 New drugs targeting the IGF family

For many years now, the gold standard of treatment for advanced or metastatic prostatic cancer has been androgen deprivation therapy (ADT) and second-generation androgen receptors signaling inhibitors (41). Unexpected resistance to castration and recurrence in the form of castration-resistant prostate cancer, for which there are few therapy choices, are regrettably major events contributing to the poor longevity of patients with prostatic cancer, even though these therapies initially demonstrated several pros (105). A lot of research has been done to design agents that target the IGF family and its signaling pathway. For instance, some studies aimed at reducing IGF-I levels or inhibiting the activity of IGF-IR and the downstream signaling pathways. Some of these drugs exhibited an anti-neoplastic activity (184, 215, 216). Antagonists of the growth hormone-releasing hormone, or growth hormone receptor (e.g., pegvisomant), and analogs of somatostatin (e.g. octreotide) resulted in a reduction of IGF-I levels (217). For instance, combinatorial therapy using octreotide and complete androgen blockage had beneficial results in patients with prostate cancer (218). Also, pegvisomant treatment halted the proliferation of prostatic cancer cells (219). As such, anti-IGF-1R monoclonal antibodies (mAbs), human neutralizing IGF antibodies, and tyrosine kinase inhibitors (ligand-gated IGF-R) have been developed. Part of these drugs have been evaluated in clinical studies regarding prostatic cancer either alone or in conjunction with traditional therapy (220).

### 8.1 Monoclonal anti-IGF-1R antibodies

The primary approach entails employing anti-IGF-1R mAbs to impede ligand-receptor interactions, thereby inducing the internalization and subsequent degradation of IGF-1R. For the treatment of malignant tumors, a number of therapeutic mAbs targeting IGF-1R have been developed, including ganitumab (IgG1) (139), cixutumumab (IgG1) (221–223), and figitumumab (IgG2) (222, 224, 225). The antibodies that have undergone evaluation in clinical trials have demonstrated anti-tumor growth effects to a certain degree and have been well tolerated by the majority of participants.

Cixutumumab and figitumumab have shown mixed results in clinical trials, with some benefits in PSA levels but inconsistent overall survival improvements (221, 226, 227). A12, another mAb, demonstrated promise in preclinical studies by inhibiting cancer cell growth, but requires further research (228, 229). Ganitumab, a unique mAb that avoids interfering with insulin signaling, showed good tolerability but lacks clinical trials in prostate cancer patients (139, 222). Other mAbs like BIIB022 are being explored in other cancers with positive safety profiles (230). Overall, IGF-1R mAbs hold promise for prostatic cancer treatment, but further research is needed to optimize their efficacy and safety, particularly in combination therapies, while exploring new mAbs with improved targeting strategies.

### 8.2 Neutralizing antibodies for IGF

Monoclonal antibodies that neutralize IGF ligands inhibit proliferative and pro-survival signaling, which are initiated by IGF ligands. IGF-I/II neutralizing monoclonal antibodies simultaneously inhibit multiple IGF signaling pathways but do not affect insulin receptor-β (INSR), which regulates glucose homeostasis (231). As a result, they do not increase the risk of hyperglycemia compared to IGF-1R/INSR tyrosine kinase inhibitors. Few neutralizing antibodies for IGF have been developed, including xentuzumab (IgG1) and dusigitumab (IgG2). Human antibody xentuzumab (BI 836845) specifically targeted IGF-I and IGF-II. It exhibited potential in the treatment of breast cancer and in the mitigation of resistance in prostate cancer. It mainly functions by inhibiting IGF-I levels and impeding its effects. The initial trials were met with acclaim (232–234). Dusigitumab (MEDI-573) is an additional IGF signaling-targeting antibody, whose metabolic impact appeared to be comparatively lesser in nature when compared to alternative IGF-targeting therapies. Although it exhibited anti-tumor properties, its efficacy might be constrained by its relatively low binding affinity (235).

### 8.3 Tyrosine kinase inhibitors of IGF-1R

Linsitinib targets the insulin and IGF-1R receptors and demonstrated promise in preclinical research but had poor efficacy in clinical trials for prostate cancer among other malignancies. Though it was well tolerated, neither the tumor response nor PSA levels were improved. Though further research is needed, linsitinib may be helpful in breaking through chemotherapy resistance (141, 236, 237). An alternative tyrosine kinase inhibitor, BMS-754807, has shown promise in preclinical research by reducing the proliferation and causing cell death in cancer cells (238). It also affected insulin receptors, though, which could have an effect on blood sugar regulation (239). Its safety and effectiveness in prostate cancer patients have not yet been evaluated in clinical trials.

## 9 Conclusions

PCa remains a public health burden. The relationship between the IGF family and prostate cancer is intricate as IGF signaling plays a significant role in the development and progression of prostate cancer. The activation of its downstream signaling pathways promotes cell proliferation, migration, and invasion. Targeting IGF signaling has been considered as a potential therapeutic strategy for PCa. Researchers have explored various approaches to inhibit IGF signaling using antibodies or small molecule inhibitors against various components of the IGF signaling pathway. Therefore, further understanding of the role of IGF signaling in PCa shows promise and needs to be considered in the context of a comprehensive treatment strategy.

## Author contributions

NE: Writing – original draft, Writing – review & editing. HH: Writing – original draft, Writing – review & editing. MS: Writing – original draft, Writing – review & editing. WE: Writing – original draft, Writing – review & editing. IT: Writing – original draft, Writing – review & editing.
